# Growing Interest in Global Health Among Trainees: The Need for Increasing Training Opportunities for Residents and Fellows in Oncology

**DOI:** 10.1155/ghe3/6095104

**Published:** 2025-07-12

**Authors:** Darya Kizub, Chidinma P. Anakwenze, Han Cun, Kathleen M. Schmeler, Cameron E. Gaskill

**Affiliations:** ^1^Department of Breast Medical Oncology, The University of Texas MD Anderson Cancer Center, Houston, Texas, USA; ^2^Department of Radiation Oncology, The University of Texas MD Anderson Cancer Center, Houston, Texas, USA; ^3^Department of Obstetrics and Gynecology, University of Chicago, Chicago, Illinois, USA; ^4^Department of Gynecologic Oncology and Reproductive Medicine, The University of Texas MD Anderson Cancer Center, Houston, Texas, USA; ^5^Department of Surgery, University of California at Davis, Sacramento, California, USA

## Abstract

**Purpose:** Disparities in Global Cancer Care outcomes continue to grow between high- and low- and middle-income countries (LMICs). Specific competencies are required to provide effective oncologic care in low-resource settings. We assessed trainee interest and participation in global oncology and training activities at a major cancer center to determine support for future global oncology program development.

**Methods:** An online survey was administered to trainees at MD Anderson Cancer Center in November 2020. Questions addressed interest in global health, prior experience, perceptions of mentorship and opportunities, career aspirations, and interest in participation in global oncology training.

**Results:** Survey links were emailed to all trainees (*n* = 318) enrolled in oncology-related residency and fellowship training programs. Completed surveys were returned by 72 trainees (22.6%) spanning 17 programs. Thirty-three trainees expressed interest in global health, and 8 (24.2%) had previous or ongoing experience specific to global oncology. Seven (21.2%) indicated they had good access to global oncology faculty mentorship, while 26 (78.8%) indicated little to no access to mentorship. Thirty (90%) indicated that they wished to include global oncology activities in their future careers. More than half of the respondents indicated interest in participating in global oncology activities in training, including collaborative research projects with partners abroad, clinical work and education abroad, and global oncology grand rounds and journal clubs. Thirteen (39.4%) were interested in a global health track and 12 (36.4%) requested formalized coursework.

**Conclusions:** We found significant interest in global health among trainees in oncology specialties at MD Anderson. As a result, the institution is implementing the Global Cancer Care Track for all trainees. The track includes a formalized curriculum, mentorship, research, and clinical opportunities to develop future leaders in global oncology with the goal of improving cancer care in low-resource settings.

## 1. Introduction

Global cancer incidence is predicted to increase by nearly 50% over the next 20 years, with a majority of cases occurring in low- and middle-income countries (LMICs) [1, 2]. Cancer prevention, diagnosis, and care delivery are complex and resource-intensive. The current personnel and supply infrastructure required to address the increasing cancer burden in LMICs is grossly deficient [3]. Medical oncologists in LMICs see an average of over twice as many new consults per year as compared to their counterparts in high-income countries [4], with less than half of LMIC providers having dependable access to essential chemotherapies [5]. In addition, it is estimated that more than 90% of cancer patients in LMICs lack access to radiotherapy [6]. The European Society of Oncology recently published a report assessing the distribution of the multidisciplinary cancer care workforce, finding LMICs fall short of benchmarked capacity across all disciplines [7].

There is growing recognition that providing cancer care in low-resource settings requires specific competencies within each specialty [8]. To address the impending shortage of medical oncologists, both the American Society of Clinical Oncology (ASCO) and the European Society of Medical Oncology (ESMO) now include a core curriculum for global health within their standards for medical oncology training [9]. Likewise, the Canadian Association of Radiation Oncology and the American Society for Radiation Oncology (CARO-ARRO) have developed a postgraduate global health competency curriculum for radiation oncology [10]. While just under half of National Cancer Institute (NCI)-designated cancer centers reported dedicated global health programs, over 80% of these had some level of global oncology training and reported trainee interest in global oncology across multiple specialties [11].

MD Anderson Cancer Center is a leading institution for multiple specialty training programs in oncology. However, at the time this study was conducted, it did not have formalized global oncology training. The purpose of our study was to survey trainees to catalog the ongoing global oncology activities across the institution and to determine trainee interest in a formalized global oncology training curriculum.

## 2. Materials and Methods

### 2.1. Setting

The survey was administered in November 2020 to participants enrolled in clinical and nonclinical oncology-related training programs at The University of Texas MD Anderson Cancer Center. The survey was distributed via email with a link to the REDCap instrument (Vanderbilt University, Nashville, TN). One reminder email was issued, and all responses were voluntary.

### 2.2. Study Design

The survey focused on 5 main themes: (1) interest in global health, (2) prior experience in global health and global oncology, (3) perceptions of available mentorship and opportunities at MD Anderson, (4) career aspirations and perceived obstacles, and (5) interest in participating in global oncology training (Appendix A).

### 2.3. Data Collection and Analysis

Survey responses were collected and aggregated using REDCap. All response data were stored on internal secure servers. Data analysis was performed in Excel (Microsoft, Redmond, WA) and Stata (StataCorp, College Station, TX).

### 2.4. Ethics

Approval for the study was granted by The University of Texas MD Anderson Cancer Center Institutional Quality Improvement Advisory Board.

## 3. Results

Ninety-one trainee programs, constituting around 318 individuals, were contacted to complete the survey. Seventy-two trainees (22.6%) from 17 programs completed the survey.

### 3.1. Interest and Experience With Global Health and Global Oncology

Thirty-three (45.8%) of the 72 trainees who completed the survey indicated an interest in global health, including seven (21.2%) from hematology/medical oncology, six (18.2%) from radiation oncology, three (9.1%) each from surgical oncology, gynecologic oncology, and pediatric oncology, two from cancer prevention research, and one each from breast surgical oncology, neurologic oncology, urologic oncology, onco-hospital medicine, cancer anesthesiology, transplant-oncology infectious disease, and investigational cancer therapeutics. Trainees had completed a mean of 1.3 years of training (range: 0.5–4.0) and had a mean of 1.9 years of training (range: 1.0–4.0) remaining.

**Figure 1:**
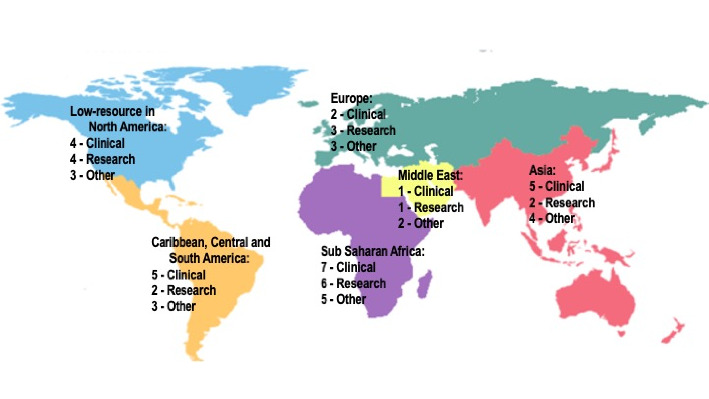
Map of respondent's prior experience in global health.

Of the 33 respondents with an interest in global health, 14 (42.4%) had no prior experience with global health. Nineteen reported some prior global health experience, including prior clinical experience (13; 39.4%), research experience (8; 24.2%), experience with advocacy (6; 18.2%), and/or general readings/staying up to date on issues (11; 33.3%). Only eight (24.2%) had experience specific to global oncology. The median number of years of global health experience was one year, with five having no experience, two having an experience of a few months, five having one year of experience, four (12.1%) having three years of experience, and one each having 2, 4, 7, 11, and 15 years of experience. Of the 20 trainees, 10 (50.0%) had done work in sub-Saharan Africa, 7 (35.5%) worked with underserved populations in North America, 5 (25.0%) worked in Latin America and the Caribbean, 3 (15%) in South Asia, and 2 (10%) in the Middle East and North Africa (Figure 1).

Respondents demonstrated varied interpretations of the definition of global oncology; of the 33 trainees interested in global health, 13 (39.4%) described global oncology as provision and study of cancer care in LMICs, while 10 (30.3%) defined it more broadly as serving underserved people around the world regardless of geography and including high-income countries. Five (15.2%) defined global oncology as any work related to cancer that involves an international partner, and 4 (12%) as multicenter/multinational studies related to cancer.

### 3.2. Mentorship and Training Opportunities

Fourteen (42.4%) knew of faculty engaged in global oncology work in their department or division. Seven (21.2%) thought that they had access to a good amount of faculty mentorship to pursue their interest in global oncology work, while 12 (36.4%) thought they had access to only a little mentorship in this regard, and 14 (42.4%) thought they had none. No trainees thought that there was extensive mentorship available in global oncology at MD Anderson.

Of the 33 trainees with an interest in global health, 21 (63.6%) responded that their global oncology experience could be improved with increased mentorship availability. Twenty-two (66.7%) desired more opportunities to work on research projects focused on global health issues and 19 (57.6%) thought that their educational experience would be enhanced by clinical experiences in LMICs. Seventeen (51.5%) thought that their global oncology experience could be enhanced by having dedicated time within their program to pursue global health activities and 18 (54.5%) desired more funding opportunities.

### 3.3. Career Aspirations and Perceived Obstacles

**Figure 2:**
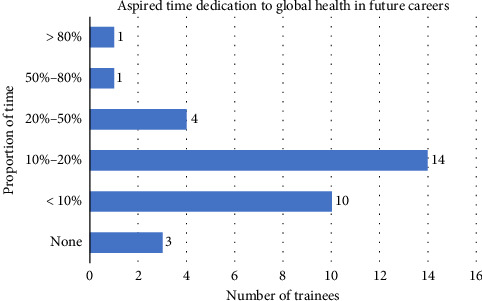
Aspired time dedication to global health in future careers.

Of the 33 respondents interested in global oncology, 30 (90%) indicated that they wished to include global oncology activities in their future careers. Of these, nearly half (14; 42.4%) intended to dedicate 10%–20% of their time to global oncology work, 10 (30.3%) intended to spend < 10% of their time, and 4 (12.1%) 20%–50% of their time. Two respondents expressed a desire to dedicate > 50% of their academic time to global oncology work (Figure 2).

Twenty-six of the 33 trainees interested in global health answered the open-ended question about their perception of the important issues in global oncology. Key themes in the responses included a focus on health equity and disparities in cancer care between LMIC and high-income countries related to cancer prevention, early detection, and access to diagnosis and treatment; importance of developing long-term and sustainable relationships with partners in LMIC; the importance of mentorship, protected time, and funding in pursuing a career in global oncology; and the negative impact of COVID-19 on current global oncology efforts (Table 1).

### 3.4. Interest in Participating in Global Oncology–Related Activities at MD Anderson

Of the 33 trainees who were interested in global health, all indicated an interest in participating in global oncology activities and training while at MD Anderson. Twenty-five (75.8%) were interested in pursuing collaborative research projects with partners in LMICs, while 21 (63.6%) each were interested in clinical work and taking part in the education or training of clinicians or researchers in LMICs. Twenty-three (69.7%) were interested in participating in in-person or virtual grand rounds or journal clubs related to global oncology. Nineteen (57.6%) each were interested in participating in in-person or virtual informal meetings/happy hours with other MD Anderson faculty and trainees interested in global oncology. Eighteen (54.5%) were interested in participating in virtual tumor boards with partners in LMICs. Twenty-seven (81.8%) expressed interest in receiving additional updates about global oncology activities at MD Anderson, and 17 (51.5%) were newly added to the HSS Global Oncology Subcommittee listserve as a result. Thirteen (39.4%) were interested in a global health track at MD Anderson, and 12 (36.4%) requested formalized global health education and/or coursework.

## 4. Discussion

This work summarizes the first survey of interest in global health and global oncology among trainees at The University of Texas MD Anderson Cancer Center. Notably, 90% of respondents wish to make global oncology a part of their future career; however, few had any prior experience with global oncology activities. Our findings highlight the keen interest of trainees across a variety of programs focused on cancer care to develop clinical, capacity-building, and research global oncology projects. However, faculty mentorship and protected time for these trainees to pursue their global health interests were perceived to be limited. Trainees were interested in additional training in global oncology, whether through attending global oncology grand rounds and journal clubs, pursuing formal coursework, or a formal global health certificate as part of their program. Most of the trainees had a mean of almost 2 years of training remaining, giving an opportunity to launch global oncology initiatives that may enrich current trainees' education.

Increased interest in global health has been documented across medical education. A 2021 survey [12] reported that 65% of medical students had an interest in a global health career, yet only 31% had participated in a global health rotation as students. A large majority of students ranked the opportunity to participate in global health as an important aspect of selecting a residency program, with only a slightly smaller proportion recognizing the importance of programs having a formalized curriculum. Similar to our results, the majority of trainees interested in participating in global oncology activities did not have prior experience.

In response to this growing interest, global health training programs and fellowships have increased in number [13–15]. Dedicated tracks in residency aim to increase trainee exposure to global health opportunities and promote scholarly activity [16]. Prior global health research and/or clinical experience has been associated with a 3-4-fold increase in medical trainees' interest in pursuing a future global health career [17]. Our results indicated that trainees hope to rely on experiences in their fellowships to prepare them to make global oncology part of their careers. Programs can harness trainee enthusiasm and provide global health experiences and training with a constructive and conscientious curriculum. Most impactfully, these programs have been shown to increase international collaboration and result in increased resources to partnering sites [18, 19]. Dedicated global health research programs are instrumental in not only increasing a trainee's research productivity in their future careers but also in the creation of future global health leaders and educators [20, 21].

Global health is also quickly being recognized as an academic subspecialty and a potential career track for trainees [22, 23]. Major academic organizations have produced guidelines for career success in global health, highlighting the need to develop education programs, create scholarly contributions, and secure research funding [24, 25]. Our results show trainees echo these priorities, emphasizing the importance of practicing global oncology with academic rigor, scholarly work, and teaching. However, in order to prepare trainees for success in their early careers, mentorship and training specific to global health are required [24, 26]. Society organizations, such as ESMO, ASCO, and CARO-ARRO, have already produced curricula and competencies in global oncology that can be readily adopted by training programs. Expansion of the global oncology curriculum is still needed in fields such as surgical oncology, surgical subspecialties, and pathology.

Prior to finalizing the conclusion of these results, several limitations warrant discussion. First, our results are based on a relatively small proportion of potential respondents. The survey response rate was only 22.6%; however, the possible self-selection of survey respondants likely represents those interested in global oncology and provides meaningful insight into the group most likely to lead such initiatives. This may bias a report of exaggerated global health enthusiasm. However, prior research suggests exposure to global health fosters continued interest and thus we believe that as global oncology initiatives grow, we will see more and more rally to these important programs. Second, while these data included a vast variety of training programs, it is limited to a single institution. This may skew results towards trainees interested in an academic career based on biases of trainees choosing to train at a large academic center and may not accurately represent the larger oncology training community. Various undergraduate training programs were contacted to complete the survey; however, respondents were all postgraduate trainees, missing important information on a large group of individuals (nursing students, surgical technology trainees, etc.) essential to global health efforts. Future studies may consider expanding the cohort to include trainees from public health, dentistry, nursing, and other health professionals to capture the full spectrum of global health interest across medicine and affiliated fields. It could potentially strengthen the case for broader institutional support of global health initiatives.

Despite these limitations, these survey results have helped to inform the programmatic development of global oncology at MD Anderson. The House Staff Senate created a specialized Global Oncology Subcommittee that now organizes monthly global oncology grand rounds in partnership with the Global Academic Programs. This subcommittee has cataloged existing faculty projects that trainees may participate in, as well as advocate for the creation of additional global oncology opportunities across campus. Efforts are also underway to increase the number of faculty with protected time devoted to global oncology and to collaborate with faculty outside of MD Anderson to increase the number of mentors available to trainees.

Furthermore, the survey results were the first step to justifying the development of an institutional global oncology curriculum, the MD Anderson Global Cancer Care (GCC) Track. This trainee-led initiative, supported by program directors, cancer center leadership, and the general medical education (GME), is a 2-year program that provides a robust educational curriculum, structured multidisciplinary mentorship, and global oncology research training to create future leaders in global oncology. The curriculum was developed in accordance with the competencies established by the ASCO Academic Global Oncology Task Force [24] and includes CARO-ARRO didactics. Upon completion of the curriculum and a clinical elective, educational project, or research project, trainees earn a certificate of Track completion at graduation. The first five fellows (2 radiation oncology trainees, 2 gynecologic oncology trainees, and 1 medical oncology trainee) were accepted into the program last year.

## 5. Conclusions

There is significant interest in global health among trainees in oncology specialties. In addition to curriculum development, trainees desire strengthened mentorship, research, and clinical opportunities. Efforts in these areas should be pursued with the hope of improving opportunities for the United States-based trainees and facilitating future work with local LMIC collaborators to address the needed cancer care in low-resource settings.

## Figures and Tables

**Table 1:** Perceived most important issues in global oncology.

Sustainable efforts in low-resource settings. Adequate prevention programs in low-resource settings
Implementation of early detection interventions known to work; working with pharmaceutical companies to make treatment more affordable; training of front-line providers to recognize signs and symptoms of cancer to improve referral; raising awareness in the general population about cancer as a survivable disease; signs and symptoms of cancer
Appropriate mentorship and identifying the right connections with other interested academics globally
Workforce creation; sustainable programs; funding long-term; impact of political landscape on programs; sustained interest of high-income nations; lack of trust of LMICs in therapies
Patients are living longer as a direct result of public health measures and better treatments for infectious diseases, thus the cancer burden in LMICs is rapidly increasing, far outpacing current treatment capacities
Care capacity including screening/diagnostics, supply chain management, and provision of care
Access to clinical trials
Lack of infrastructure; awareness and governmental support; education and training
Access
Disparities between treatment options offered in LMIC versus HIC
Lack of resources
Technologic disparities
Outreach
Inequitable access to care
Access to care; timeliness of care
Disparities
Cultural challenges
Differences in healthcare systems and infrastructure across the globe. Differences in access to care
Access to resources in rural areas (in both high- and low-income countries)
Funding and time off from training to attend the global health oncology initiative
COVID-19 restrictions for travel; dedicated time for trainees to get involved so that they can carry this forward into their careers; centers that are consistent in their care for low-income countries instead of only short-term missions; funding is of course always a hurdle
The COVID-19 pandemic and the limitations in travel; funding; time with regards to participating
Setting up a mentorship or training program with the infrastructure to provide longevity of the transfer of ideas, and clinical and research collaboration
Funding; COVID-19-related travel restrictions; lack of senior mentorship in global oncology
COVID-19
Do not know

## Data Availability

The data that support the findings of this study are available from the corresponding author upon reasonable request.

## Appendix A. Survey Instrument

A brief survey on House Staff interest and experience with global oncology (GCC across all disciplines of cancer including medical oncology, surgery, radiology, pathology, gynecology, anesthesia, plastics, research, education, palliative care, spiritual care, public health, and others).

This survey will take 5 min or less to complete.

1. Do you have an interest in global health? (Choose one)
   (A) Yes
   (B) No
   (C) Maybe
^∗^(If no, end survey; If yes/maybe, then the survey goes on)
2. What is your prior experience with global health? (Check all that apply)
   (A) None
   (B) Clinical
   (C) Research
   (D) Advocacy
   (E) Read about it and stay abreast of happenings (as much as I can)
   (F) Other: Free text
  (2b) (If B-F above, then) How many years of experience do you have with global health work?
   #
  (2c) (If B-F above, then) What geographic areas does your global health work involve? (Check all that apply)
   (A) East Asia and Pacific
   (B) Europe and Central Asia
   (C) Latin America and the Caribbean
   (D) Middle East and North Africa
   (E) South Asia
   (F) Sub-Saharan Africa
   (G) Underprivileged populations in North America
  (2d) (If B-F above, then) Do you have prior/current experience with global oncology? (Choose one)
   (A) Yes
   (B) No
3. Does your department/division at MD Anderson have faculty who are engaged in global oncology work? (Choose one)
   (A) Yes
   (B) No
   (C) Not sure
4. Do you feel that you have the mentorship to pursue your global oncology interest? (Choose one)
   (A) None
   (B) A little (somewhat)
   (C) A good amount
   (D) Extensive
5. Where is this mentorship based? (Choose one)
   (A) None, N/A
   (B) MD Anderson
   (C) Outside institution
   (D) Both MD Anderson and an outside institution
6. Are you interested in participating in the following global oncology activities at MD Anderson? (Check all that apply)
   (A) Clinical work in LMICs
   (B) Collaborative research projects with partners in LMICs
   (C) Education/training of clinicians or researchers in LMICs
   (D) Participation in multidisciplinary tumor boards with partners in LMICs
   (E) In-person or virtual lectures/grand rounds or journal clubs related to global oncology
   (F) In-person or virtual informal meetings/happy hours with other MD Anderson faculty/trainees interested in global oncology
   (G) Other: Free text
7. What resources and/or opportunities at MD Anderson do you believe would enhance your global oncology experience? (Check all that apply)
   (A) Mentorship
   (B) Clinical experiences in LMICs
   (C) Research projects focused on global health issues
   (D) Formalized global health education and/or coursework
   (E) Dedicated time within your program to pursue global health activities
   (G) Formalized track/certification in global health within your program
   (H) Funding opportunities
   (I) Other: Free text
8. How much time would you like to spend on global oncology activities during your career? (Choose one)
   (A) < 10%
   (B) 10%–20%
   (C) 20%–50%
   (D) 50%–80%
   (E) > 80%
   (F) Unsure
9. What statement best describes your present or desired work in global oncology? (Choose one)
   (A) Cancer work involving an international partner (regardless of nature or work or where it is being done)
   (B) The study of cancer and provision of cancer care to underserved people around the world regardless of geography-including high-income countries
   (C) The study of cancer and provision of cancer care to people in LMICs.
   (D) Multicenter/multinational research studies related to cancer
   (E) Other: Free text
10. What do you think are the biggest issues in global oncology at present? (Free text)
11. Please provide the following information:
  Name (First name Last name)
   (Free text)
  Fellowship/residency program
   (Free text)
  How many years have you been in training at MD Anderson?
   #
  Number of training years remaining at MD Anderson? (If you graduate in 2021, write 1 year)
   #
12. Please provide your email below if you are interested in receiving information about global health activities at MD Anderson (free text).
